# Disseminated Nocardiosis in a Renal Transplant Recipient

**DOI:** 10.7759/cureus.12497

**Published:** 2021-01-05

**Authors:** Mohamedanwar Ghandour, Hammam Shereef, Hassan Homida, Sanjay Revankar, Mareena S Zachariah

**Affiliations:** 1 Internal Medicine/Nephrology, Wayne State University Detroit Medical Center, Detroit, USA; 2 Internal Medicine, Beaumont Health, Dearborn, USA; 3 Internal Medicine, Advocate Aurora Health Care, Detroit, USA; 4 Infectious Diseases, Wayne State University Detroit Medical Center, Detroit, USA; 5 Nephrology, Wayne State University School of Medicine, Detroit, USA

**Keywords:** disseminated nocardiosis, renal transplant

## Abstract

Nocardiosis is an uncommon opportunistic Gram-positive bacterial infection caused by aerobic actinomycetes in the genus *Nocardia*. *Nocardia* can cause localized or systemic suppurative diseases involving eyes, kidneys, skin, lungs, bone, and central nervous system. Disseminated nocardiosis is a rare condition, seen among immunocompromised patients. We report the case of a 55-year-old African American, kidney transplant male recipient on maintenance immunosuppression, who was diagnosed with cutaneous and pulmonary nocardiosis. Presenting symptoms were shortness of breath, and bilateral lower extremities pain and swelling. Tissue culture grew Gram-positive bacilli specified as *Nocardia farcinica* from thigh and gluteal abscesses. CT thorax showed bilateral reticulonodular opacities. The patient was managed with immunosuppression reduction and specific treatment with high-dose trimethoprim-sulfamethoxazole (TMP-SMX) in conjunction with linezolid. Combination antibiotics were continued for four weeks, and thereafter, TMP-SMX alone was continued for 12 months, at which point all lesions had healed. Nocardiosis with systemic involvement carries a poor prognosis. However, early diagnosis and appropriate antibiotic coverage had a favorable outcome in a renal transplant recipient. Recommended treatment duration is 6 to 12 months.

## Introduction

Nocardiosis is an uncommon opportunistic Gram-positive bacterial infection caused by aerobic actinomycetes in the genus *Nocardia*. It can cause localized or systemic suppurative diseases in humans, involving eyes, kidneys, skin, pulmonary, brain as well as disseminated infection. Inhalation of the organism is the most common mode of entry; that is why the majority of infections involve lungs. Herein, we report a case of a renal transplant recipient who presented with disseminated nocardiosis with pulmonary and cutaneous involvement and was successfully treated with combined antibiotic therapy and surgical drainage.

## Case presentation

We report the case of a 55-year-old male with a past medical history of end-stage kidney disease secondary to medical renal disease of hypertension. After a dialysis vintage of six years, he received a deceased donor renal transplant in 2018. Post-transplant, his allograft function remained stable with a baseline serum creatinine level at 1.6-1.8 mg/dL. After induction with basiliximab, the patient was maintained on triple immunosuppressive therapy consisting of mycophenolic acid 720 mg twice daily per oral (PO), tacrolimus 2 mg twice daily PO, and prednisone 5 mg daily PO.

Six months following the renal transplant, the patient presented to the hospital with a chief complaint of progressive bilateral lower extremities pain with swelling, which started 10 days before presentation. Associated symptoms included shortness of breath. Workup included an ultrasound of the right lower leg, which showed a heterogeneous mass in the right posterior thigh; nevertheless, the patient received hydrocodone/acetaminophen and was instructed to follow up with his primary care physician in a week. However, one week later, the patient presented again due to the worsening of symptoms; physical exam revealed a palpable, fluctuant, and tender fullness involving the left gluteal and the right posterior thigh region with associated left knee swelling.

**Figure 1:**
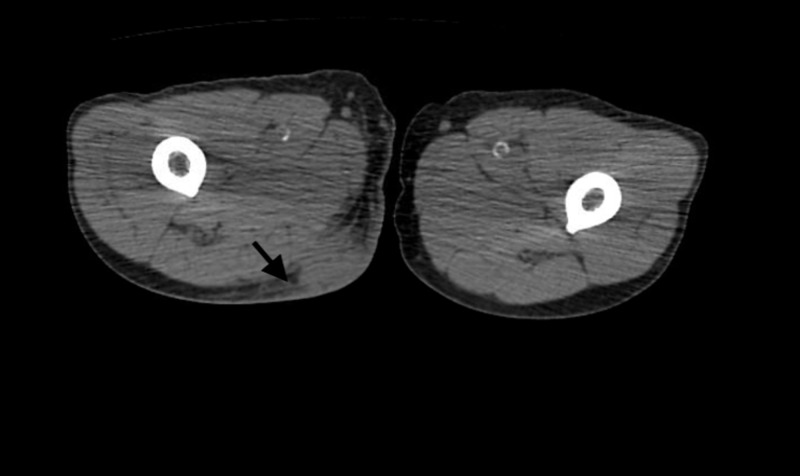
CT pelvis and bilateral lower extremities without contrast showed a 3.5-cm fluid collection (arrow) in the right posterior thigh

**Figure 2:**
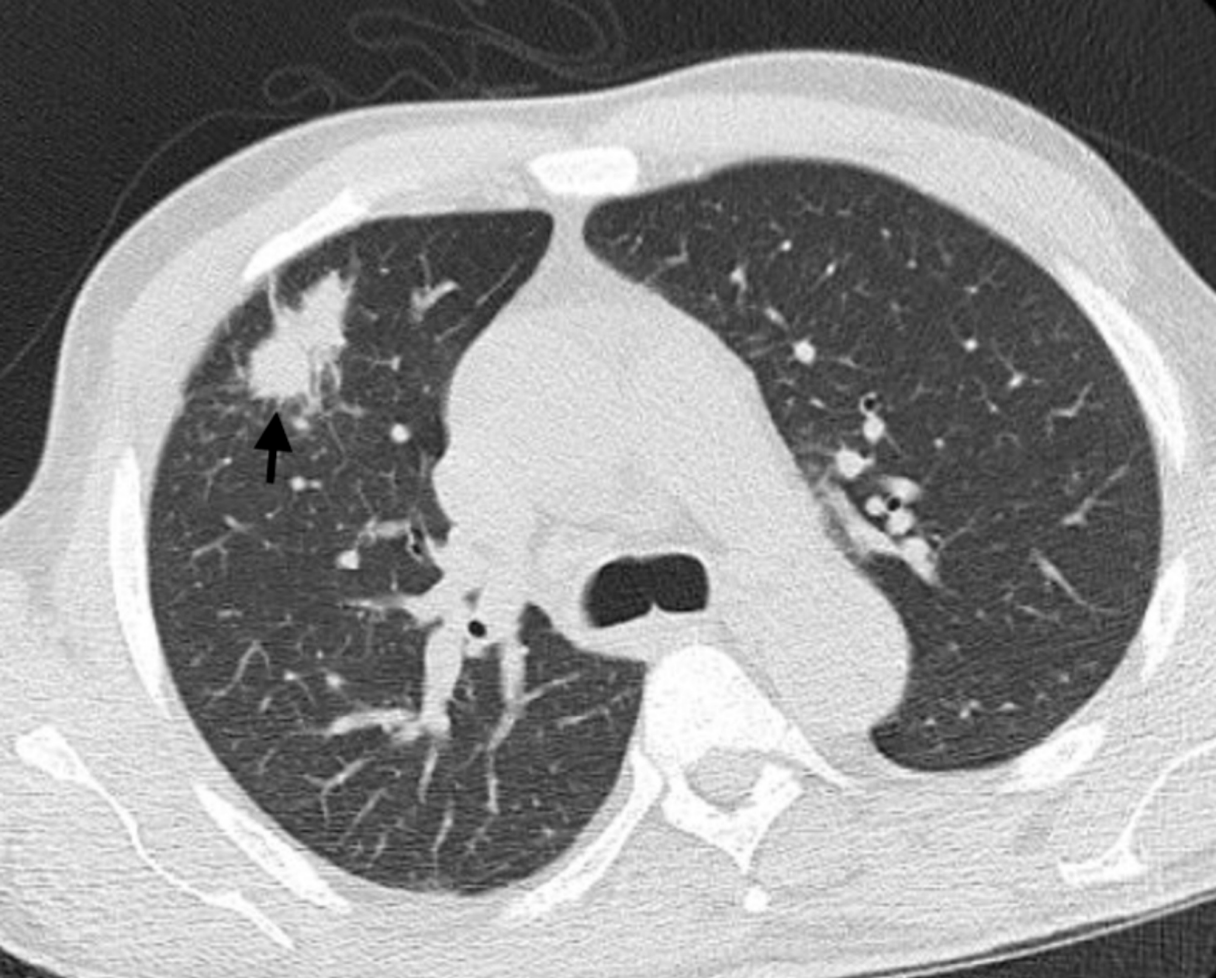
CT thorax showing pulmonary nodule (arrow)

Laboratory workup revealed the following: C-reactive protein 149 mg/L, creatinine 1.65 mg/dL (baseline creatinine 1.6-1.8 mg/dL), and white blood cell count 9,500/mm^3^. CT pelvis and bilateral lower extremities without contrast showed a 3.5-cm fluid collection in the right posterior thigh and the left buttock (Figure 1). Chest CT showed multi-focal irregular airspace opacities with new scattered sub-centimeter pulmonary nodules (Figure 2). The two-dimensional echo was negative for valve vegetations. MRI brain was negative for masses or abscesses; blood cultures were negative and tissue culture grew Gram-positive bacilli identified as *Nocardia farcinica*. The patient underwent incision and drainage of the thigh and gluteal abscesses with the placement of Penrose drain and surgical site cultures obtained. Tacrolimus and myfortic acid were held for two weeks due to the presumed infectious process; prednisone 20 mg daily PO was initiated along with high-dose trimethoprim-sulfamethoxazole (TMP-SMX) in conjunction with linezolid. The patient’s condition subsequently improved and he was discharged on oral linezolid and TMP-SMX. He was treated with the combination for four weeks, and then continued with TMP-SMX alone for a total of 12 months at which point all lesions had resolved without allograft dysfunction.

## Discussion

Renal transplant patients are at an increased risk of certain infections, particularly following the initiation of immunosuppressive drugs after the transplant [1]. Nocardia infection is commonly seen among patients with T-cell and macrophage function disorders, such as patients who are taking immunosuppressive agents [2]. The risk of nocardiosis is increased in the first year following renal transplantation, presumably due to using immunosuppression to prevent rejection [3]. Patients treated with steroid-sparing regimens, such as cyclosporine, have found significantly reduced rates of nocardial infections, to 0.7%, in renal transplant recipients [4]. The patient was diagnosed with nocardiosis six months following the renal transplant.

*Nocardia* species are not ordinarily seen in the respiratory tract, yet pulmonary involvement is the primary site of nocardial infection in more than two-thirds of cases [3,5]. Therefore, a sputum culture is mostly indicative of *Nocardia* infection. Rarely, respiratory nocardial isolate has been considered a non-pathogen (i.e., colonizer) [6]. Most pulmonary infections are primary, but *Nocardia* can spread to the lung from other sites, such as the skin [7]. On the other hand, cutaneous lesions are reported in kidney transplant recipients with a *Nocardia* infection [2]. Manifestations of primary cutaneous nocardiosis include ulcerations, pyoderma, cellulitis, nodules, and subcutaneous abscesses [8,9]. The reported patient had disseminated nocardiosis involving lungs and skin, though lungs were thought to be the primary source of infection. Also, imaging findings in pulmonary nocardiosis are different, including lung nodules, lung masses (with or without cavitation), reticulonodular infiltrates, interstitial infiltrates, lobar consolidation, subpleural plaques, and pleural effusions [10]. Our patient’s CT thorax showed irregular airspace opacities in the left upper lobe.

TMP-SMX is considered first-line therapy for nocardiosis. However, some *Nocardia* species are resistant to TMP-SMX, including *N. farcinica*, while *Nocardia asteroides* and *Nocardia nova* remain sensitive primarily to TMP-SMX, with susceptibility rates of >95% and 89%, respectively [11,12]. Moreover, there is no consensus for the role of TMP-SMX as prophylaxis for *Nocardia*. Although one study proposed that TMP-SMX prophylaxis is effective in heart transplant recipients, nocardiosis has been documented in one-third of the renal transplant recipients who received TMP-SMX as *Pneumocystis carinii* prophylaxis [13]. Deaths related to *Nocardia* infection in transplant recipients have been rare. In one study, the nocardial infection has been neither found to be associated with mortality nor overall survival. Also, in the same study, early diagnosis suggests a favorable therapeutic outcome [14].

## Conclusions

Overall, disseminated nocardiosis with systemic involvement carries a grave prognosis. However, early diagnosis of the case, with proper antibiotic coverage, leads to a favorable outcome, even in a post-renal transplant patient.
